# Supplementary material for: Detection and Molecular Phylogenetic-Morphometric Characterization of Rhizoctonia tuliparum, Causal Agent of Gray Bulb Rot of Tulips and Bulbous Iris
Source: J Fungi (Basel). 2022 Feb 8;8(2):163. doi: 10.3390/jof8020163 (PMC8880388; doi:10.3390/jof8020163)
Supplement: Supplementary file 1 [file jof-08-00163-s001.zip › Figure S1.pdf]

Figure S1. Selected *Rhizoctonia*, *Waitea* and *Ceratobasidium* sequences from the alignment file for the ITS region for designing primers and probes for the quantitative PCR diagnostic assay for detection and quantification of *Rhizoctonia tuliparum*. Only the sequences of *Rhizoctonia tuliparum* (synonym *Sclerotium tuliparum*) showed the presence of all three primers.

(Forward primer – Probe – Reverse primer sequences are highlighted

>AF308621. Rhizoctonia solani, AG2-2 WB, isolate A4
CAATTTGGTTTCACTTTGGTATTGGAGGTTCTTGCAGCTTCACATGCTGC
TCCTCTTTGTTCATTAGCTGGATCTCGGTGTTATGCTTGGTTCCACTCGG

>AB122124. Rhizoctonia solani, AG 2-1, isolate B-1
CAATTGGTTTCACTTTGGTATTGGAGGTCTTTTGCAGCTTCACACCTGCT

CCTCTTTGTGCATTAGCTGGATCTCAGTGTTATGCTTGGTTCCACTCGG

>AB054849. Rhizoctonia solani, AG-2-1, isolate 88-033
CAATTGGTTTCACTTTGGTATTGGAGGTCTTTTGCAGCTTCACACCTGCT
CCTCTTTGTTCATTAGCTGGATCTCAGTGTTATGCTTGGTTCCACTCGG

>DQ356411. Rhizoctonia solani, AG-2-1, isolate Rh 120208
CAATTGGTTTCACTTTGGTATTGGAGGTCTTTTGCAGCTTCACACCTGCT

CCTCTTTGTTCATTAGCTGGATCTCAGTGTTATGCTTGGTTCCACTCGG

>DQ356412. Rhizoctonia solani, AG-2-1, isolate Rh 1202325
CAATTGGTTTCACTTTGGTATTGGAGGTCTTTTGCAGCTTCACACCTGCT
CCTCTTTGTTCATTAGCTGGATCTCAGTGTTATGCTTGGTTCCACTCGG

>AF354105. Rhizoctonia solani, AG-2-1, isolate 56Rs
CAATTGGTTTCACTTTGGTATTGGAGGTCTTTTGCAGCTTCACACCTGCT

CCTCTTTGTTCATTAGCTGGATCTCAGTGTTATGCTTGGTTCCACTCGG

>AF354063. Rhizoctonia solani, AG-2-1, isolate 8Rs
CAATTGGTTTCACTTTGGTATTGGAGGTCTTTTGCAGCTTCACACCTGCT
CCTCTTTGTTCATTAGCTGGATCTCAGTGTTATGCTTGGTTCCACTCGG

>DQ356408. Rhizoctonia solani, AG-10, isolate Rh 100278
CAATTGGGTTGACTTTGGTATTGGAGGTCTTGCAGGTTCACACTTGCTCC

TCTTTGTGCATTAGCTGGATCTCAGTGTTATGCTTGGTTCCACTCGG

>AY154318. Rhizoctonia solani, AG-10
CAATTGGGTTGACTTTGGTATTGGAGGTCTTGCAGGTTCACACTTGCTCC
TCTTTGTGCATTAGCTGGATCTCAGTGTTATGCTTGGTTCCACTCGG

>AB019026. Rhizoctonia solani, AG-10, isolate Rh 76107
CAATTGGGTTGACTTTGGTATTGGAGGTCTTGCAGGTTCACACTTGCTCC
TCTTTGTGCATTAGCTGGATCTCAGTGTTATGCTTGGTTCCACTCGG

>DQ356410. Rhizoctonia solani, AG-10, isolate Rh 120203
CAATTGGGTTGACTTTGGTATTGGAGGTCTTGCAGGTTCACACTTGCTCC

TCTTTGTGCATTAGCTGGATCTCAGTGTTATGCTTGGTTCCACTCGG

>AF153800. Rhizoctonia solani, AG-10, isolate 93017
CAATTGGGTTGACTTTGGTATTGGAGGTCTTGCAGGTTCACACTTGCTCC

TCTTTGTGCATTAGCTGGATCTCAGTGTTATGCTTGGTTCCACTCGG

>DQ356413. Rhizoctonia solani, AG-8, isolate Rh 070304
CAATTGGTTCTACTTTGGTATTGGAGGTCTTTTGCAGCTTCACACCTGCT
CCTCTTTGTGCATTAGCTGGATCTCAGTGTTATGCTTGGTTCCACTCAG

>AF354067. Rhizoctonia solani, AG-8, isolate (ZG1-2)SA50
CAATTGGTTCTACTTTGGTATTGGAGGTCTTTTGCAGCTTCACACCTGCT

CCTCTTTGTGCATTAGCTGGATCTCAGTGTTATGCTTGGTTCCACTCAG

>AF354068. Rhizoctonia solani, AG-8, isolate (ZG1-3)SA1512
CAATTGGTTCTACTTTGGTATTTGGAGGTCTTTTGCAGCTTCACACCTGC

TCCTCTTTGTGCATTAGCTGGATCTCAGTGTTATGCTTGGTTCCACTCAT

>KX767078. Sclerotium tuliparum, isolate I-399
CGAGGTCGACTTTTTGTTTTGGACTTGGAGGCCTTTGCGGATTCACGTCC
GCTCCTCTCAAACACATTAGCTGGATCTCAGTGAACTCGGTTCCACTCGG

>KX767079. Sclerotium tuliparum, isolate DGF2-VV
CGAGGTCGACTTTTTGTTTTGGACTTGGAGGCCTTTGCGGATTCACGTCC
GCTCCTCTCAAACACATTAGCTGGATCTCAGTGAACTCGGTTCCACTCGG

>KX767080. Sclerotium tuliparum, isolate DGF3-Car
CGAGGTCGACTTTTTGTTTTGGACTTGGAGGCCTTTGCGGATTCACGTCC
GCTCCTCTCAAACACATTAGCTGGATCTCAGTGAACTCGGTTCCACTCGG

>EU191041. Sclerotium tuliparum, isolate 29792
CGAGGTCGACTTTTTGTTTTGGACTTGGAGGCCTTTGCGGATTCACGTCC
GCTCCTCTCAAACACATTAGCTGGATCTCAGTGAACTCGGTTCCACTCGG

>MH854847. Sclerotium tuliparum, strain CBS 206.25
CGAGGTCGACTTTTTGTTTTGGACTTGGAGGCCTTTGCGGATTCACGTCC
CCTCCTCTCAAACACATTAGCTGGATCTCAGTGAACTCGGTTCCCCTCGG

>DQ102426. Ceratobasidium sp. AG-A isolate Str75
CAATTGGCCTTGCTTTGGATTTGGAGGTCTTGCAGATGTCACAGTCTGCT
CCTCTTAAATGAATTAGCTGGATCTCAGTATACACTTGGTTCCACTCGG

>AJ242882. Rhizoctonia sp., strain Eab-S2
CGATTGATTTTATTTTGGACTTGGGGGTCTGCAGATTCACGTCTGCTCCC
CTTAAATTCATTAGCTGGATCACTGTAGACTCGGTTCCACTCGG

>AJ242884. Rhizoctonia sp., strain Eab-S4
CGATTGATTTTATTTTGGACTTGGGGGTCTGCAGATTCACGTCTGCTCCC
CTTAAATTCATTAGCTGGATCACTGTAGACTCGGTTCCACTCGG

>DQ102442. Ceratobasidium sp. AG-I, isolate Ibs1
CGATCGATTTTATTTTGGACTTGGAGGTCTGCAGATTCACGTCTGCTCCT
CTTAAATTCATTAGCTGGATCTGTATGAACTCGGTTCCACTCGG

>AJ419932. Rhizoctonia sp., strain AV-2
CGATCGATTTTATTTTGGACTTGGAGGTCTGCAGATTCACGTCTGCTCCT
CTTAAATTCATTAGCTGGATCTGTATGAACTCGGTTCCACTCGG

>AB196650. Ceratobasidium sp. AG-I, strain AV-2
CGATCGATTTTATTTTGGACTTGGAGGTCTGCAGATTCACGTCTGCTCCT
CTTAAATTCATTAGCTGGATCTGTATGAACTCGGTTCCACTCGG

>DQ356407. Uncultured Ceratobasidiaceae, isolate Rh100249
CGATTGGTTTTATTTTGGACTTGGGGGTCTGCAGATTCACGTCTGCTCCC
CTTAAATTCATTAGCTGGATCACTGTAGACTCGGTTCCACTCGG

>DQ356409. Uncultured Ceratobasidiaceae, isolate Rh 1202334
CGATTGGTTTTATCTTGGACTTGGGGGTCTGCAGATTCACGTCTGCTCCC
CTTAAATTCATTAGCTGGATCACTGTAGACTCGGTTCCACTCGG

>AB213577. Waitea circinata var. agrostis, isolate TOM-BFW
AATTGTTGTGTTTGGACTTGGAAGCTGTTGGCGCAAGTCAACTCTTCTTA

AATGTATTAGCTGGGGTTTATATAGTTGGATCCTTGG

>AB213596. Waitea circinata var. zeae, isolate Rz-1-sh
AATTATTGTGTTTGGACTTGGAAGCTTGTCGGCGCAAGTCGACTCTTCTT

AAATGCATTAGCTGGGGTTTATATAGTTGGATCCTTGG

>AB213591. Waitea circinata var. oryzae, isolate Ro119: Aichi
GAATCATTATTATTTGGACTTGGAAGTTTGTCGGCGCAAGTCGACTCTTC

TTAAATGCATTAGCTGGGGTTTATATAGTTGGATCCTTGG

>DQ356414. Waitea circinate, isolate Rh 120232
GTTTTTGTGTTTGGACTTGGAAGCTGTCGGCGCAAGTCGACTCTTCTTAA

ATGCATTAGCTGGGGTTTATATAGTGGATCCTTGG

>DQ356415. Waitea circinate, isolate Ro4-4
TTAATTGTTGTATTTGGACTTGGAAGCTGTTGGCGCAAGTCGACTCTTCT

CAAATTCATTAGCTGGGGTTATATAGTTGGATCCTTGG

>AB213584. Waitea circinata var. circinata, isolate AMI-BG
GATTGTTGTATTTGGACTTGGAAGCTGTTGGCGCAAGTCGACTCTTCTCA

AATGTATTAGCTGGGGTTTATATAGTTGGATCCTTGG

>AF222799. Waitea circinate, isolate RZ01
GATTGTTGTATTTGGACTTGGAAGCTGTTGGCGCAAGTCGACTCTTCTCA

AATGTATTAGCTGGGGTTTATATAAGTTGGATCCTTGG

>DQ356416. Waitea circinate, isolate Rh 0801344
GATTGTTGTATTTGGACTTGGAAGCTGTTGGCACAAGTCGACTCTTCTCA

AATGTATTAGCTGGGGTTTATATAGTTGGATCCTTGG

>DQ356417. Waitea circinate, isolate Rh 0801387
GATTGTTGTATTTGGACTTGGAAGCTGTTGGCGCAAGTCGACTCTTCTCA

AATGTATTAGCTGGGGTTTATATAGTTGGATCCTTGG

>MH855251. Ceratobasidium anceps, strain CBS 152.32
CGAGGTCGACTTTTGTTTTGGACTTGGAGGCTTGCAGATTTCACGTTTGC

TCCTCTCAAATAAATTAGCTGGATCTCCGTGAACTCGGTTCCACTCGG

>AJ427402. Ceratobasidium anceps, isolate CBS 152.32
CGAGGTCGACTTTTGTTTTGGACTTGGAGGCTTGCAGATTTCACGTTTGC

TCCTCTCAAATAAATTAGCTGGATCTCCGTGAACTCGGTTCCACTCGG

>KF386035, Rhizoctonia butinii, isolate 11026
CAACTGGTTTTGTTTTGGACTTGGAGGTCTTGCGGATTCACGTCTGCTCC

TCTTAAAAATATTAGCTGGATCTCAGTGAACTCGGTTCCACTCGG

>MH855815, Rhizoctonia floccose, strain CBS 336.36
CGGCTGACTTTGTTTTGGACTTGGAGGTCTTGCAGATTCACGTCTGCTCC

TCTTAAAAGCATTAGCTGGATCTCAGTGAGCTCGGTTCCACTCGG
