# Supplementary material for: Detection and Molecular Phylogenetic-Morphometric Characterization of Rhizoctonia tuliparum, Causal Agent of Gray Bulb Rot of Tulips and Bulbous Iris
Source: J Fungi (Basel). 2022 Feb 8;8(2):163. doi: 10.3390/jof8020163 (PMC8880388; doi:10.3390/jof8020163)

Figure S2. *Rhizoctonia tuliparum* isolated from bulbous iris and grown on potato dextrose agar produced "lab-grown" sclerotia that were slightly different in appearance (left) from a mixture of "natural light" and "natural dark" sclerotia of *R. tuliparum* (right) that were collected directly from soil around diseased iris bulbs.

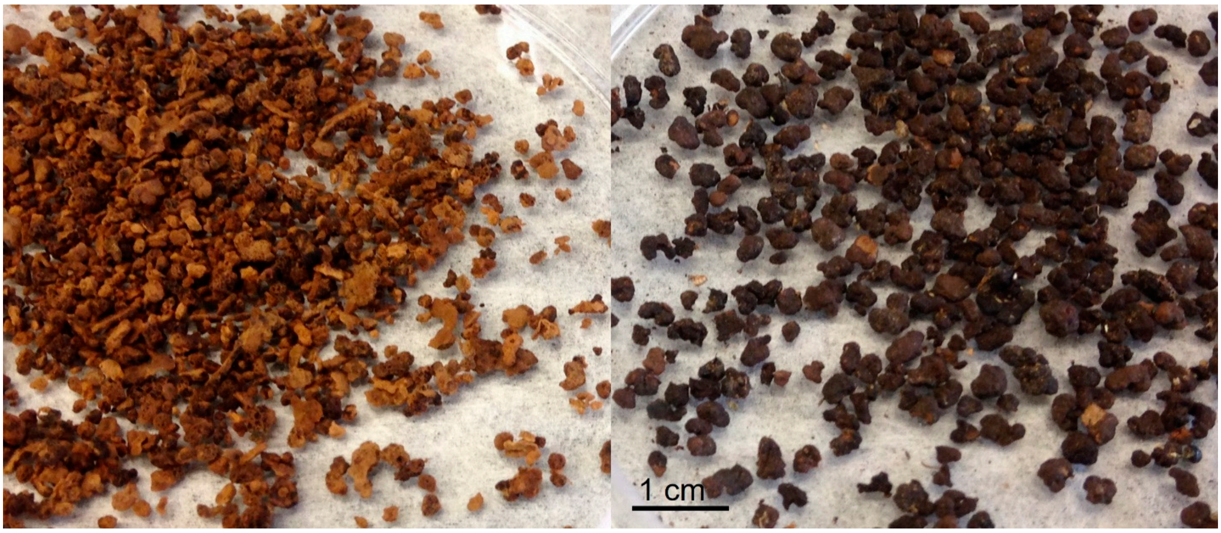

Supplement: Supplementary file 1 [file jof-08-00163-s001.zip › Figure S2.pdf]
