# Supplementary material for: Detection and Molecular Phylogenetic-Morphometric Characterization of Rhizoctonia tuliparum, Causal Agent of Gray Bulb Rot of Tulips and Bulbous Iris
Source: J Fungi (Basel). 2022 Feb 8;8(2):163. doi: 10.3390/jof8020163 (PMC8880388; doi:10.3390/jof8020163)
Supplement: Supplementary file 1 [file jof-08-00163-s001.zip › Table S1.pdf]

Table S1. The ITS2 sequence length and base composition of *C. anceps* and three *R. tuliparum* isolates.

	**Bases**	**A**	**A%**	**T**	**T%**	**G**	**G%**	**C**	**C%**	**GC%**
MH855251 (*C. anceps*)	237	66	27	70	31	51	21	50	21	42.6
KX767078 (Washington state)	238	60	25	68	30	53	22	57	23	46.2
EU191041 (Europe)	238	61	25	68	30	53	22	56	23	45.8
MH854847 (Whetzel)	238	62	26	67	30	52	21	57	23	45.8
