# Supplementary material for: Detection and Molecular Phylogenetic-Morphometric Characterization of Rhizoctonia tuliparum, Causal Agent of Gray Bulb Rot of Tulips and Bulbous Iris
Source: J Fungi (Basel). 2022 Feb 8;8(2):163. doi: 10.3390/jof8020163 (PMC8880388; doi:10.3390/jof8020163)
Supplement: Supplementary file 1 [file jof-08-00163-s001.zip › Table S2.pdf]

Table S2. Base-pairing in the ITS2 RNA secondary structures of varieties of *R. tuliparum* Washington isolate (KX767078) in comparison to that of *Ceratobasidium anceps* (MH855251).

	**Mutation in Helix I**	**Effect on helix I**	**Mutation in Helix II**	**Effect on helix II**	**Mutation in Helix III**	**Effect on helix III**	**Mutation in Helix IV**	**Effect on helix IV**
KX767078 (Washington)		No gain/loss of base pairing	A-U → G-C	CBC	Loss of one U-A and two G-C pairs	Net loss of three base pairs	U-A deletion	Loss of one base pair
			G-U → G-C	hCBC	Maintained the pseudoknot structure			
