# Supplementary material for: Detection and Molecular Phylogenetic-Morphometric Characterization of Rhizoctonia tuliparum, Causal Agent of Gray Bulb Rot of Tulips and Bulbous Iris
Source: J Fungi (Basel). 2022 Feb 8;8(2):163. doi: 10.3390/jof8020163 (PMC8880388; doi:10.3390/jof8020163)
Supplement: Supplementary file 1 [file jof-08-00163-s001.zip › Table S3.pdf]

Table S3. The conserved terminal loop sequences in all isolates of *Ceratobasidium anceps* (MH855251 and AJ427402), and *Rhizoctonia tuliparum* (KX767078, EU191041, and MH854847).

	Loop I	Loop II	Loop III	Loop IV
Conserved Sequences in *C. anceps* & *R. tuliparum*	AUU	CAC	AAGU	UAG
