# Supplementary material for: Detection and Molecular Phylogenetic-Morphometric Characterization of Rhizoctonia tuliparum, Causal Agent of Gray Bulb Rot of Tulips and Bulbous Iris
Source: J Fungi (Basel). 2022 Feb 8;8(2):163. doi: 10.3390/jof8020163 (PMC8880388; doi:10.3390/jof8020163)
Supplement: Supplementary file 1 [file jof-08-00163-s001.zip › Table S4.pdf]

Table S4. Base-pairing in the ITS2 RNA secondary structures and the conserved terminal loop sequences of the European (EU191041) and Whetzel (MH854847) isolates of *R. tuliparum* with respect to the Washington isolate (KX767078).

	**Mutation in Helix I**	**Effect on helix I**	**Mutation in Helix II**	**Effect on helix II**	**Mutation in Helix III**	**Effect on helix III**	**Mutation in Helix IV**	**Effect on helix IV**
EU191041 (European)	None	No change	None	No change	C → A (In the loop region)	Gain of an A-U base pair	None	No change
MH854847 (Whetzel)	None	No change	G → C	Loss of a C-G base pair	C → A (In the loop region)	Gain of an A-U base pair	G → A	Loss of a C-G base pair
					A → C (pseudoknot-forming region)	Loss of an A-U base pair needed to form pseudoknot		
					C → G	Loss of a C-G base pair		
