# Supplementary material for: Detection and Molecular Phylogenetic-Morphometric Characterization of Rhizoctonia tuliparum, Causal Agent of Gray Bulb Rot of Tulips and Bulbous Iris
Source: J Fungi (Basel). 2022 Feb 8;8(2):163. doi: 10.3390/jof8020163 (PMC8880388; doi:10.3390/jof8020163)
Supplement: Supplementary file 1 [file jof-08-00163-s001.zip › Table S5.pdf]

Table S5. Presence or absence of pseudoknot structures in helix III of *Ceratobasidium* binucleate and uninucleate isolates within the greater clade containing *Rhizoctonia tuliparum* in Fig. 6.

Anastomosis Group*	GenBank Accession No.	Pseudoknot present (+) / absent (-)	AG group$
A	DQ102417	+	1
Ba	AB286930	+	5
Bb	AB122144	+	5
B(o)	AB219143	+	5
C	AB290021	+	6
D (*C. cereale*)	JQ768017	+	4
E	AF354083	-	3
Fa	DQ102434	-	3
Fb	AB219145	-	3
G	DQ102395	+	2
H	AF354089	+	6
I	AB290022	+	6
K	AB286932	-	1
L	AB286934	+	2
O	AF354094	-	2
P	AB286940	-	3
Q	AF354095	+	5
R	AB219146	-	3
S	AF354084	-	3
U	HQ269825	-	3
UNR1*	AF472295	+	
UNR1*	AF472278	+	
UNR2*	AF200515	-	
UNR2*	AF200517	-	
C. anceps@	MH855251	+	
C. fluccosa@	MH855815	+	
R. alpina@	MH855685	+	
R. butini@	KP334098	+	
C. pseudocornigerum@	MH861653	+	
C. noxium@	EU810056	+	

*Uninucleate *Ceratobasidium* isolate group 1 and group 2 per Sharon et al., (2008); $Per Sharon et al. (2008), AG group 1: -A and -K; AG group 2: -G, -L, and -O; AG group 3: -E, -F, -P (and -U), -R, and -S; AG group 4: AG-D; AG group 5: -B (including its three subgroups) and -Q; AG group 6: -C, -H, and -I; *Ceratobasidium* species grouped in a larger clade containing the *R. tuliparum* isolates.
