# Supplementary material for: Detection and Molecular Phylogenetic-Morphometric Characterization of Rhizoctonia tuliparum, Causal Agent of Gray Bulb Rot of Tulips and Bulbous Iris
Source: J Fungi (Basel). 2022 Feb 8;8(2):163. doi: 10.3390/jof8020163 (PMC8880388; doi:10.3390/jof8020163)
Supplement: Supplementary file 1 [file jof-08-00163-s001.zip › Table S6.pdf]

Table S6. GenBank Accessions of the ITS (ITS1-5.8S-ITS2) regions showing complete sequence match with the Q-PCR Probe* to detect *Rhizoctonia tuliparum*.

GenBank Accession	Genus/species	Isolate/strain /clone	Source/host	Country
MH854847	*Sclerotium tuliparum*	CBS 206.25	Tulip	NY, USA
EU191041	*S. tuliparum*	29792	Iris	Netherlands
KX767078	*S. tuliparum*	I-399	Iris	WA, USA
KX767079	*S. tuliparum*	DGF2-VV	Iris	WA, USA
KX767080	*S. tuliparum*	DGF3-Car	Iris	WA, USA
LC597348	Ceratobasidiaceae sp.	CE22_F499	*Phalaenopsis japonica*	Japan
MT236771	Uncultured fungus	4248_679	irrigation water from the pond	Lithuania
MH861653	*Ceratobasidium pseudocornigerum*	CBS 568.83	*Pterostylis mutica*	Australia
NR_154601	*C. angustisporum*	CBS 568.83	*P. mutica*	Australia
LC384936	*Rhizoctonia* sp. (*Ceratobasidium* sp.)	MAFF 411008	*Fagus crenata*	Japan
MG252920	Uncultured Ceratobasidiaceae	OUT_22	*Gymnadenia conopsea*	China
MW854608	*Ceratobasidium* sp.	OTU_P	*P. scabra*_W217	Australia
MW854582	*Ceratobasidium* sp.	OUT_P	*P. mutica*_QLD12	Australia
MN006068	*Ceratobasidium* sp.	OTU_275	*Gymnadenia conopsea*	Australia
MN006062	*Ceratobasidium* sp.	OUT_177	*G. conopsea*	China
MW854581	*Ceratobasidium* sp.	OUT P	*Pterostylis mutica QLD11*	Australia
MW854580	*Ceratobasidium* sp.	OUT P	*P. mutica QLD9*	Australia
MW854560	*Ceratobasidium* sp.	OUT P	*P. dolichochila Pdolich*	Australia

**Rtul* Probe TTTGCGGATTCACGTCC
